# From Bench to Bedside in Precision Medicine: Diabetes Mellitus and Peri-Implantitis Clinical Indices with a Short-Term Follow-Up: A Systematic Review and Meta-Analysis

**DOI:** 10.3390/jpm12020235

**Published:** 2022-02-08

**Authors:** Mario Dioguardi, Stefania Cantore, Salvatore Scacco, Cristian Quarta, Diego Sovereto, Francesca Spirito, Mario Alovisi, Giuseppe Troiano, Riccardo Aiuto, Daniele Garcovich, Vito Crincoli, Luigi Laino, Michele Covelli, Annarita Malcangi, Lorenzo Lo Muzio, Andrea Ballini, Michele Di Cosola

**Affiliations:** 1 Department of Clinical and Experimental Medicine, University of Foggia, Via Rovelli 50, 71122 Foggia, Italy; mario.dioguardi@unifg.it (M.D.); cristian_quarta.549474@unifg.it (C.Q.); diego_sovereto.546709@unifg.it (D.S.); spirito.francesca97@gmail.com (F.S.); giuseppe.troiano@unifg.it (G.T.); lorenzo.lomuzio@unifg.it (L.L.M.); michele.dicosola@unifg.it (M.D.C.); 2 Department of Basic Medical Sciences, Neurosciences and Sensory Organs, University of Bari “Aldo Moro”, 70124 Bari, Italy; vito.crincoli@uniba.it; 3 Faculty of Dentistry (Fakulteti i Mjekësisë Dentare-FMD), University of Medicine, 1001 Tirana, Albania; 4 Department of Surgical Sciences, Dental School, University of Turin, 10127 Turin, Italy; mario.alovisi@unito.it; 5 Department of Biomedical, Surgical, and Dental Science, University of Milan, 20122 Milan, Italy; riccardo.aiuto@unimi.it; 6 Department of Dentistry, Universidad Europea de Valencia, Paseo de la Alameda 7, 46010 Valencia, Spain; daniele.garcovich@universidadeuropea.es; 7 Multidisciplinary Department of Medical-Surgical and Odontostomatological Specialties, University of Campania “Luigi Vanvitelli”, 80121 Naples, Italy; luigi.laino@unicampania.it; 8 Interuniversity Research Center “Population, Environment and Heath”—CIRPAS, University of Bari “Aldo Moro”, 70124 Bari, Italy; dott.covelli.michele@gmail.com; 9 Public Local Health Company (Azienda Sanitaria Locale, ASL), B.A.T, 76125 Trani, Italy; annarita.malcangi@gmail.com; 10 School of Medicine, University of Bari “Aldo Moro”, 70124 Bari, Italy; 11 Department of Precision Medicine, University of Campania “Luigi Vanvitelli”, 80138 Naples, Italy

**Keywords:** diabetes mellitus, peri-implantitis, dental implants, marginal bone loss, plaque index, bleeding on probing, pocket depth, clinical biochemistry, glycosylated hemoglobin HbA1c, translational research

## Abstract

*Background and objective*: Diabetes mellitus (DM) refers to a group of metabolic disorders characterized by hyperglycemia resulting from impaired secretion or action of insulin. The high levels of glucose in the blood can negatively affect the healing processes through alterations in vascularization, bone remodeling, and with increased susceptibility to infections. Diabetes mellitus is therefore a risk factor not only for many systemic diseases, but also for localized problems such as peri-implantitis. The objective of this systematic review was to identify a clear relationship between peri-implant inflammation indices and glycemic levels, through the investigation of prospective studies that report data on a short-term follow-up period. Our hypothesis was that peri-implant inflammatory indices may already present themselves in a statistically significant way as altered in patients with DM compared to patients without DM. *Materials and methods*: This review was reported according to the Preferred Reporting Items for Systematic Review and Meta-Analysis (PRISMA). *Results*: More than 992 records were identified in the PubMed, Scopus, and Cochrane Central Register of Controlled Trial electronic databases and only seven studies were included in the meta-analysis. The results of the meta-analysis report worse outcomes in patients with DM, even in the short period of six months, for peri-implatitis inflammation indices, such as Marginal bone loss (standardized (Std). mean difference (MD) 12\6 months 0.81 [0.45, 1.17]\1.82 [0.53, 3.10]), Bleeding on probing (Std. MD 12\6 months 2.84 [1.34, 4.34]\3.44 [1.41, 5.50]), Probing depth (Std. MD 12\6 months 1.14 [0.60, 1.68]\2.24 [0.66, 3.83]), and the plaque index (Std. MD 12 months 2.83 [0.09, 5.57]). *Conclusion*: The literature linking glycaemic control to peri-implant disease is highly heterogeneous due to lack of consistency of the definition of peri-implantitis and its clinical indicators among studies. Therefore, interpretation of finding and relevance to clinical practice should be considered on individual bases. In the era of personalized medicine, the clinician should utilize individualized information from translational researches and analyze all risk factors to provide the patient with evidence-based treatment options.

## 1. Introduction

In patients who have lost teeth, and want to restore proper chewing function, dental implants provide a valid solution to replace missing teeth. 

The success rates of dental implants, according to the latest systematic reviews, are around 97.5% with a three-year follow-up period [1]. Nevertheless, there are a number of factors that can negatively affect the short and long-term survival of implants, including smoking [2,3], poor oral hygiene [4], periodontal disease [5,6], and local/systemic diseases such as diabetes [7,8] or osteoporosis [9,10,11].

Diabetes mellitus (DM) refers to a group of metabolic disorders, characterized by hyperglycemia resulting from impaired insulin secretion or action [12,13]. In fact, the most important regulator of glucose uptake from the blood is the hormone insulin, which is produced by pancreatic beta cells that act on insulin receptors to promote nutrient absorption and processing. Type 1 DM, results from loss of pancreas beta cells, caused by an autoimmune response. On the other hand, Type 2 DM begins with insulin resistance, a condition in which cells fail to respond to insulin properly: the most common cause is a combination of excessive body weight and insufficient exercise. Finally, gestational diabetes is the third main form, and occurs when pregnant women without a previous history of diabetes develop high blood sugar levels [14,15,16].

Therefore, a decrease in insulin secretion or sensitivity can cause diabetes. The chronic hyperglycemia of diabetes mellitus is associated with long-term injury such as dysfunction and failure of different organs, principally the eyes, and kidneys. In particular, excessive renal glucose reabsorption has been recognized as one of the main pathophysiological disorders in type 2 DM and a specific class of hypoglycemic agents SGLT2 inhibitors is now widely used to mitigate this phenomenon [14], in nerves, heart, and blood vessels [15,16,17,18]. It therefore represents a risk factor for many systemic and local pathologies including peri-implantitis [19,20,21].

Healing processes can be adversely affected by high blood glucose levels, with alterations in vascularity, bone remodeling, and increased susceptibility to infections leading to the conclusion that hyperglycemia has negative effects on bone remodeling and subsequent implant integration [22,23].

Studies on the survival rate of implants on patients with DM, show that there is a higher failure rate in this group of patients with a marginal bone loss higher than in patients without DM.

In fact a recent retrospective study performed by Lorean et al. (2021) with a follow-up period of five to seven years concludes that patients with elevated values of glycosylated hemoglobin (HbA1c) (8.1% to 10.0%) had greater marginal bone loss than those with lower HbA1c values, recommending the use of removable prostheses in patients with poor glycemic control [24].

A recent systematic review conducted by Tan et al. (2021) was investigated, by comparing peri-implant indices such as marginal bone loss (MBL), bleeding on probing (BOP), probing depth (PD), and plaque index (PI), in patients with diabetes and in patients without DM, with a follow-up at 12, 24, and 36 months, also performing an analysis of the subgroups and a meta regression as a function of glycosylated hemoglobin levels [25].

The meta-analysis performed by Tan et al. (2021) identified a possible dose-response trend of worsening of peri-implantitis indices such as BOP and MBL in association with glycemic control, while remaining the failure rate in the short term similar in the two groups of patients [25].

Health professionals’ engagement in translational health and medical research (HMR) is fundamental to evidence-based practice leading to better patient health outcomes.

Therefore, the present systematic review aims to seek a clear relationship between peri-implantitis inflammation indices and glycemic levels by investigating prospective studies in a short-term period of 6 to 12 months as follow-up.

The review question was consequently formulated according to the PICOS scheme: (P)opulation: Patients with diabetes; (I)ntervention: implant treatment of at least one dental element; (C)ontrol: patients without DM who have received an implant treatment; (O)utcome: Mean difference in peri-implantitis indices (MBL, BOP, PD, PI) after a short follow-up period of 6 to 12 months, (S)tudies: prospective clinical studies.

## 2. Materials and Methods 

### 2.1. Protocol and Registration

The following systematic review was reported according to the Preferred Reporting Items for Systematic Review and Meta-Analysis (PRISMA). The systematic revision was registered on PROSPERO with ID: CRD42021285400 on 16 November 2021.

### 2.2. Eligibility Criteria

All clinical studies that investigated peri-implant inflammation indices (MBL, BOP, PD, PI) in a diabetic population with the presence of a control group of non-diabetic patients, were considered potentially eligible, there had to be a follow-up of short period 6–12 months in which the indices had to be measured. The inclusion criteria were: the presence of a control group of non-diabetics, the measurement of at least one peri-implant inflammation index, a follow-up period with control at 6 months or 12 months. The exclusion criteria were all studies with high risk of bias, written not in English, and published before the year 1980. It was decided to consider only articles written after the yeas 1990, because dental implantology has undergone significant changes in form and methodology over the past 30 years, including prior studies would have generated a potential risk of bias.

### 2.3. Sources of Information

The studies were searched through the use of medical electronic databases such as SCOPUS, PubMed, and Cochrane Central Register of Controlled Trial, with restriction limits relating to the English language and to the year of publication (after 1990). The bibliographic search conducted on these 3 databases was performed on 16 October 2021 and a last update of the bibliography was performed on 7 November 2021 in search of any studies published during the drafting of the manuscript, a manual search was also conducted by bibliography of previous systematic reviews on the topic, and searches were also conducted on gray literature through Google scholar, Open gray (http://www.opengrey.eu) on 28 October 2021. The search was conducted by 3 investigators (M.D., S.C., and A.B.).

### 2.4. Research, Study Selection, Data Collection Process, and Data Characteristics

The research methodology was carried out in 4 phases.

In the first phase, the method of identifying the records was chosen taking into account the following points:

1. Choice of the number of reviewers (I) and reviewers with the task of identifying the records (II) and reviewer with the task of resolving situations of disagreement (III);
2. Choice of databases, providers and the use of gray literature;
3. Choice of keywords and combinations of words to be used;
4. Decision on the inclusion and exclusion criteria;
5. Type of data to be extracted and methods of extraction.

The second phase involved identifying the records on the databases (duplicate results were removed using the EndNote 9 software, the overlaps of studies that could not be uploaded to EndNote were manually removed after the screening phase). Screening of potentially suitable articles (through the analysis of the title and abstract) and the choice of articles to be included in the meta-analysis.

The third phase involved the comparison of the studies identified by the 3 independent reviewers and the choice of articles to be included in the systematic review (the k-agreement between the 3 reviewers was approximately 0.85).

The fourth phase involved data extraction, conducted independently by the 3 reviewers, with subsequent comparison of the extracted data. The data extracted from each study concerned the number of patients in the two groups, the number of implants placed with diameter and length, the HbA1c value with range values for each group, gender, age, follow-up, the index values of peri-implant inflammation (MBL, BOP, PD, PI) with mean and range or standard deviation (SD), the type of study, the bibliographic reference.

In this phase it was decided that there were criteria to be able to perform a meta-analysis and a subgroup analysis.

**Figure 1:**
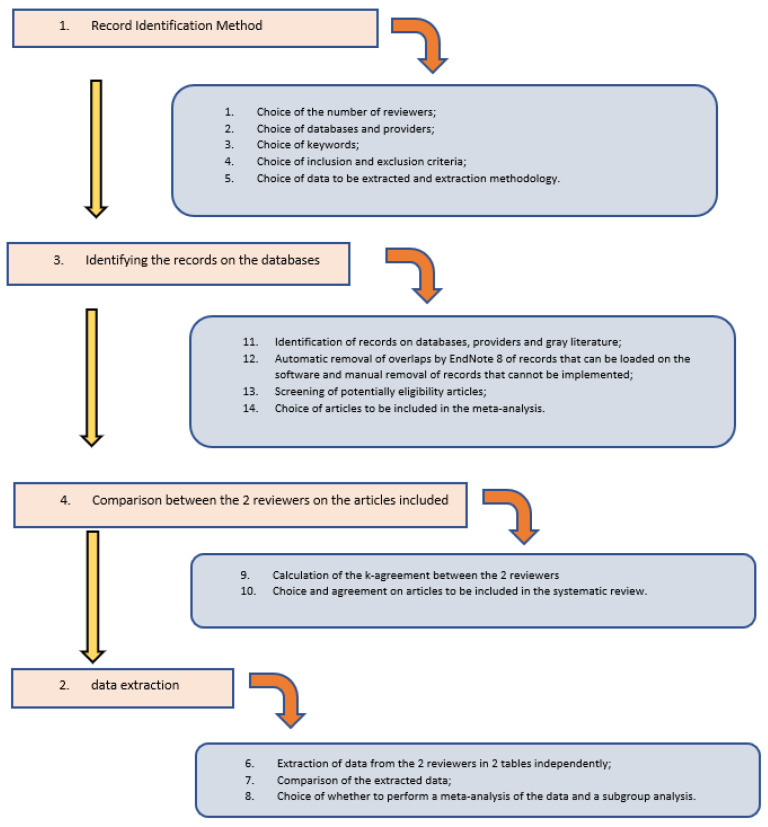
Scheme and complete protocol of the 4 phases for the selection and extraction of data, decided before the start of the search of the records. The entire protocol has been registered and approved by PROSPERO with the registration number that can be consulted: ID: CRD42021285400.

The complete research and screening methodology has been amply represented in Table 1 and Figure 1.

### 2.5. Risk of Bias in Individual Studies, Summary Measures, Summary of Results, Risk of Bias between Studies, Additional Measures

The risk of bias in the individual studies were assessed by an Author (M.D.) with a second and third author with the task of checking the correct assessment (S.C. and A.B.), for the assessment the New Castle Ottawa scale was used, furthermore it was decided, to exclude from the meta-analysis, studies that presented high risk of bias. Given the absence of heterogeneity of the measurement scales inherent in peri-implant inflammation indices, it was decided to use the mean difference of the studies between the experimental (patients with diabetes—DM) and controls (patients without diabetes—Non-DM) groups for all outcomes. The results were represented by forest plots, and inconsistency indices, such as the Higgins *I*^2^ index, were evaluated.

The risk of bias between the studies was assessed: graphically by analyzing the confidence intervals in the different forest plots; through the *I*^2^ inconsistency index (an *I*^2^ value greater than 75% was considered high and a random effects analysis was applied in specific cases); through funnel plot. If the meta-analysis presented indices of high heterogeneity, a sensitivity analysis was conducted if necessary, excluding only the studies that presented a low overlap of the confidence intervals or that graphically emerged from the Funnel Plot. Subgroup analysis was conducted as a function of HbA1c levels, but a meta-regression was not conducted. The software Reviewer Manager 5.4 (Cochrane Collaboration, Copenhagen, Denmark) was used for the meta-analysis, and in particular for mean difference, while the subgroup analysis was performed with the Open Meta-Analyst version 10 (Tufts University, Medford, MA, USA). The present study, was conducted according the GRADE pro-Guideline Development Tool online software (GRADEpro GDT, Evidence Prime, Hamilton, ON, USA) to evaluate the quality of evidence.

## 3. Results

### 3.1. Selection of Studies

**Figure 2:**
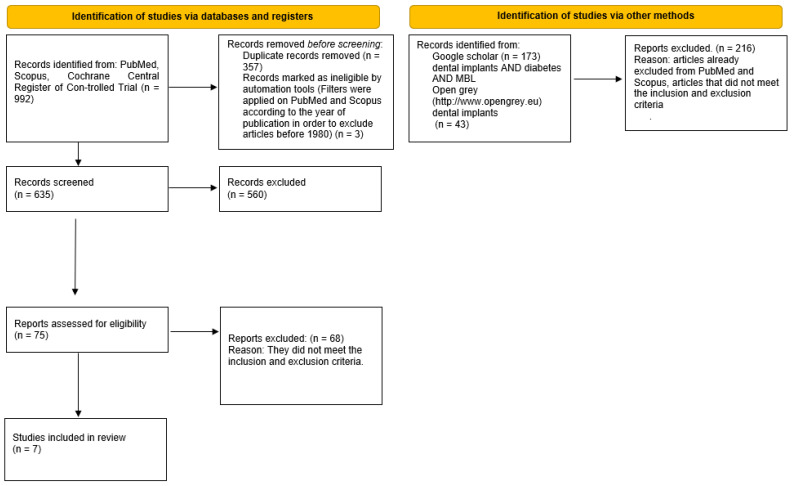
Flow chart of the different phases of the review.

The search in the PubMed, Scopus, and Cochrane Central Register of Controlled Trial databases provided 992 bibliographic sources, with the exclusion of the overlaps, 635 records were obtained. However, 560 records were excluded because, reading the abstracts, it emerged that they did not meet the eligibility criteria. The potentially eligible articles were 75, but only seven met the inclusion and exclusion criteria and were included in the meta-analysis. Besides the gray literature analysis was performed through Google scholar, Open gray (http://www.opengrey.eu, access date on 7 December 2021). No additional articles from these two databases were included (Figure 2).

### 3.2. Data Characteristics

Articles were included in the meta-analysis as follows: Tatarakis et al. (2014) [26], Gómez-Moreno et al. (2015) [27], Erdogan et al. (2015) [28], Aguilar-Salvatierra et al. (2016) [29], Cabrera-Domínguez et al. (2020) [30], Al Amri et al. (2016) [31], Alsahhaf et al. (2019) [32].

The data extracted included study design, maximum follow-up time period performed, mean age with SD or range, number of patients, gender, clinical biochemical HbA1c% levels with range or SD, number of implants placed with diameter and length, the months of follow-up to which the data refer, Plaque Index (%), Probing Depth (mm), Bleeding on Probing (%) (range or SD), and MBL in millimeters (range or SD) (Table 2).

All the seven included studies were prospective clinical studies whose results were published between 2014 and 2021 and featured a maximum three-year follow-up period. The total number of patients with diabetes (DM) recruited for the included studies was 280, and 166 for the control group (Non-DM-patients without diabetes), with a maximum mean age for the study of Tatarakis et al. (2014) [26] group DM 65 ± 8.9 (51–80) and minimum for the study of Alsahhaf et al. [32] (2019) Non-DM group 43.4 (33–49). The total number of implants placed in the DM groups was 343 while for the Non-DM group it was 212. The studies that report data for a follow-up period of only six months were three: Aguilar-Salvatierra et al., 2016 [29], Cabrera-Domínguez et al., 2020, [30], and Al Amri et al., 2016 [31]. All included studies report marginal bone loss as given.

The main outcome researched were Mean difference in peri-implantitis indices (MBL, BOP, PD, and PI) between patients with diabetes (DM) and patients without diabetes (Non-DM) with follow-up periods of 6–12 months (short-term period), was subdivided to facilitate the meta-analysis of the data (presenting diverse characteristics) in different outcomes:

1. Primary outcome: Standardized (Std) mean differences (MD) between MBL of the DM and non-DM groups at 12 months;
2. Secondary outcome: Standardized mean differences between MBL in the DM and non-DM groups at six months;
3. Tertiary outcome: Standardized mean differences between BOP of the DM and non-DM groups at 12 months;
4. Quaternary outcome: Standardized mean differences between DM and non-DM groups at six months;
5. Quinary outcome: Standardized mean differences between DM and non-DM at 12 months;
6. Senary outcome: Standardized mean differences between PD of DM and non-DM groups at six months;
7. Septenary outcome: Standardized mean differences between PI between DM and non-DM groups at 6–12 months.

### 3.3. Risk of Bias in Studies

The results of the risk of bias were reported detailed in Table 3. A value from one to three was assigned for each category (one = low and three = high).

### 3.4. Primary and Secondary Outcome (Standardized (Std). Mean Difference (MD) between MBL of the DM and Non-DM Groups at 12 Months and 6 Months)

**Figure 3:**
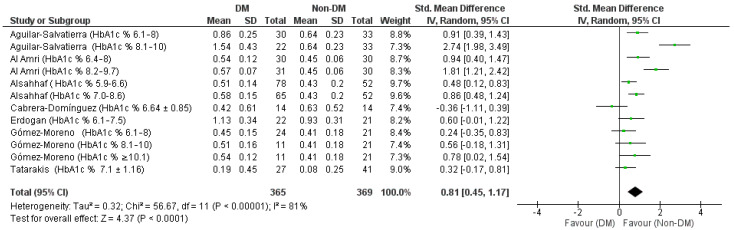
Forest plot of the random effects model of the meta-analysis of the first outcome; mean in the forest plot table, is the value of marginal bone loss between baseline and 12-month follow-up, and its standard deviation, as reported by the included studies and extracted in Table 2.

The results of the meta-analysis for the first outcome represented by the forest plot in Figure 3 show an aggregate Std. Mean Difference of 0.81 [0.45, 1.17] in support of the non-DM group, heterogeneity was high Chi² = 56.67, df = 11 (*p* < 0.00001); *I*² = 81; test for overall effect: Z = 4.37 (*p* < 0.0001).

**Figure 4:**
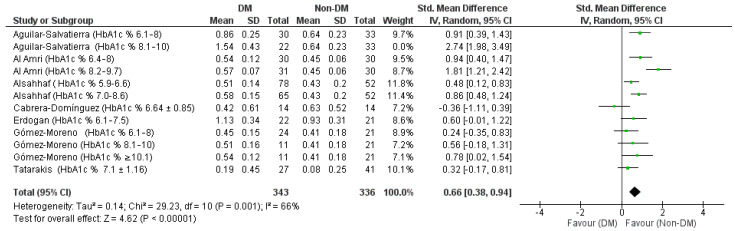
Sensitivity analysis. Forest plot of the random effects model of the meta-analysis of the first outcome, exclusion of Aguilar-Salvatierra data (HbA1c% range: 8.1–10).

Through a sensitivity analysis, by graphically evaluating the overlapping of the confidence intervals graphically from the forest plot, the data from the Aguilar-Salvatierra study (HbA1c% range: 8.1–10) was identified as a source of heterogeneity, in fact, excluding this study, heterogeneity drops to 66% (Figure 4). 

**Figure 5:**
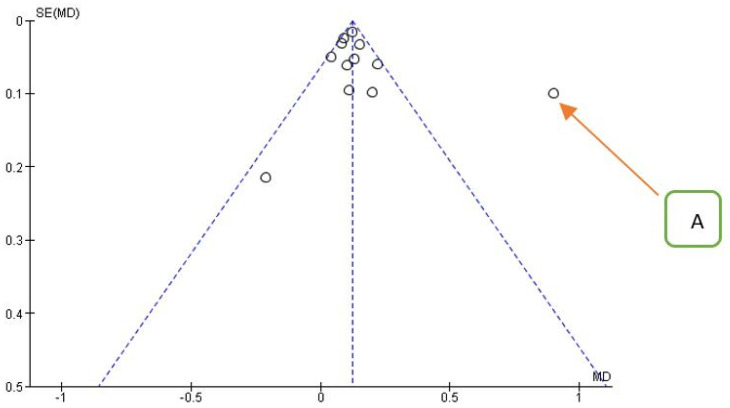
Funnel plot for the primary outcome, *I*^2^ = 81%. The presence of heterogeneity is highlighted graphically. The arrow indicates that the data of Aguilar-Salvatierra (HbA1c% range: 8.1–10) is the likely source of heterogeneity.

Confirmation of the source of heterogeneity also comes from the funnel plot for the primary outcome (Figure 5).

**Figure 6:**
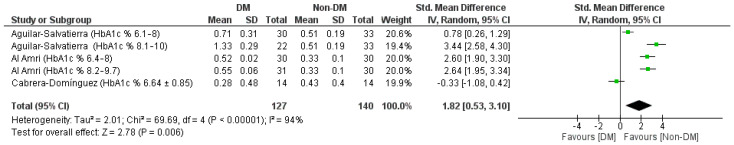
Forest plot of the random effects model of the meta-analysis of the second outcome.

**Figure 7:**
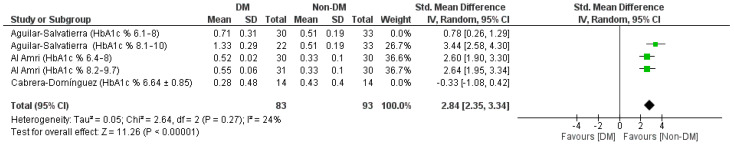
Sensitivity analysis. Forest plot of the random effects model of the secondary outcome meta-analysis, exclusion of Aguilar-Salvatierra (HbA1c% range: 8.1–10), and Cabrera-Domínguez (HbA1c% range: 6.64 ± 0.85).

The results in detail were Chi² = 29.23, df = 10 (*p* = 0.001); *I*^2^ = 66% Test for overall effect: Z = 4.62 (*p* < 0.00001) with an aggregate Std. mean difference of 0.66 [0.38, 0.94] always in favor for the Non-DM group. The data for the secondary outcome depicted in Figure 6 and Figure 7 shows heterogeneity between the data with an *I*^2^ index of 95%, also in this case the source of the heterogeneity is the data from the Aguilar-Salvatierra study [29] (HbA1c% range: 8.1–10) and Cabrera-Domínguez study (HbA1c% range: 6.64 ± 0.85) in fact, excluding this study, the heterogeneity drops to 24% (Figure 6 and Figure 7).

**Figure 8:**
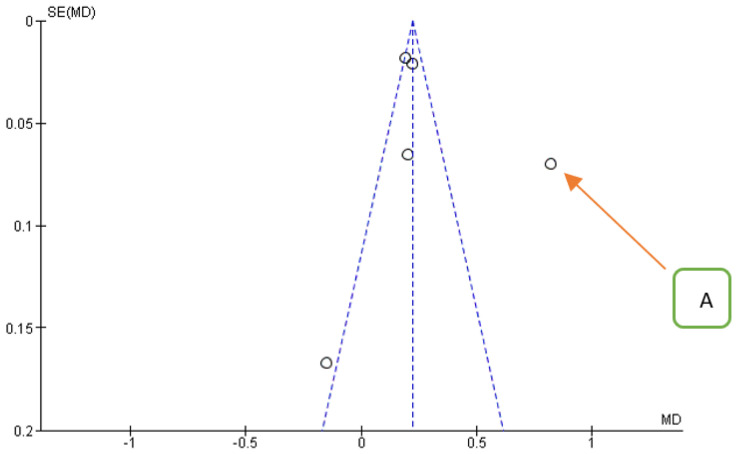
Funnel plot for the secondary outcome, *I*^2^ = 94%. The presence of heterogeneity is highlighted graphically. The arrow indicates that the data of Aguilar-Salvatierra (HbA1c% range: 8.1–10) is the likely source of heterogeneity.

Given the source of heterogeneity, one might think of a bias between studies regarding the measurement of MBL. For the second outcome Std. mean difference aggregated of 1.82 [0.53, 3.10] in favor of the non-DM group, the heterogeneity is high Chi² = 69.69, df = 4 (*p* < 0.00001); *I*^2^ = 94; test for overall effect: Z = 2.78 (*p* < 0.00001), with the exclusion of the Aguilar-Salvatierra study data (HbA1c% range: 8.1–10) and Cabrera-Domínguez (HbA1c% values: 6.64 ± 0.85) we have: Heterogeneity: Chi² = 2.64, df = 3 (*p* = 0.27); *I*^2^ = 24% Test for overall effect: Z = 11.26 (*p* < 0.00001) with an aggregate mean difference of 2.84 [2.35, 3.3] always in favor for the Non-DM group (Figure 8).

### 3.5. Tertiary and Quaternary Outcome (Std. Heterogeneity Was High and a Random MD between BOP of the DM and Non-DM Groups at 12 Months and 6 Months)

**Figure 9:**
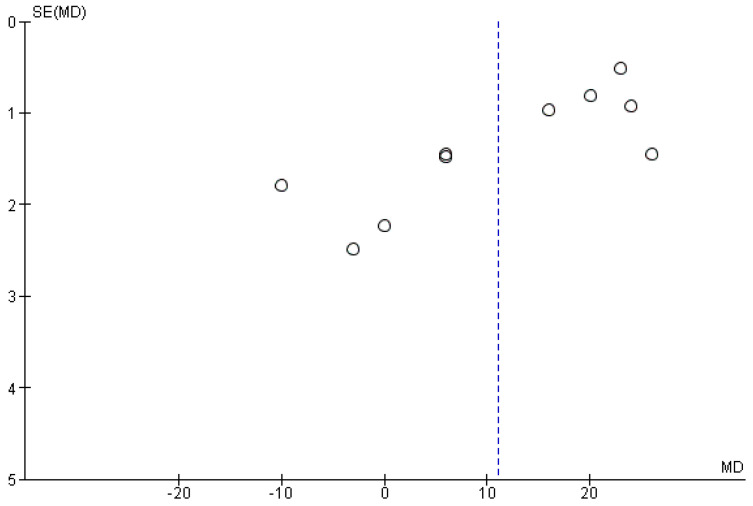
Funnel plot for the tertiary outcome, *I*^2^ = 98%, it is not possible to highlight a single source of data heterogeneity.

**Figure 10:**
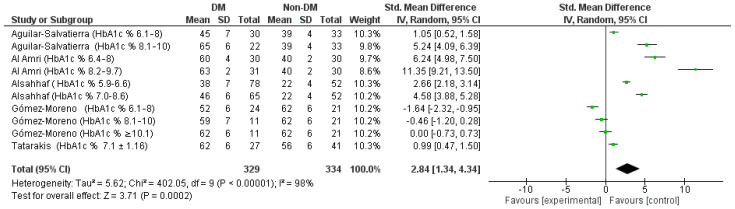
Forest plot of the random effects model of the meta-analysis of the tertiary outcome.

The meta-analysis of the data showing the BOP highlights a high heterogeneity between the studies, with the sensitivity analysis there is no possibility of highlighting the source data of the heterogeneity as also highlighted by the funnel plot (Figure 9), it is therefore decided, considering the *I*^2^ index equal to 98%, to apply a model with random effects (Figure 10).

The results of the aggregate Std. mean difference of the BOP at 12 months were identified as follows: 2.84 [3, 4.34] in support of the non-DM group, the heterogeneity is high Chi² = 402.05, df = 9 (*p* < 0.00001); *I*^2^ = 98%; Tau^2^ = 5.62 Test for overall effect: Z = 3.71 (*p* = 0.0002).

**Figure 11:**
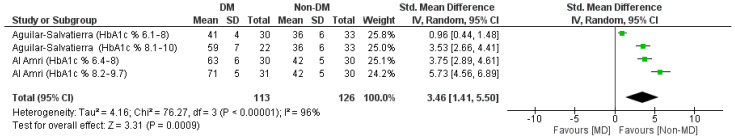
Forest plot of the random effects model of the meta-analysis of the quaternary outcome.

The meta-analysis of the quaternary outcome (Std. mean difference of the BOP with a six-month follow-up) are the following: 3.44 [1.41, 5.50] in favor of the non-DM group, heterogeneity was high and random effects have been applied in fact the values of Tau^2^ are 4.16 Chi² = 76.27, df = 3 (*p* < 0.00001); *I*^2^ = 96%; test for overall effect: Z = 3.31 (*p* = 0.0009), as show in Figure 11.

### 3.6. Quinary and Senary Outcome (Std. MD between DM and Non-DM at 12 Months and 6 Months)

**Figure 12:**
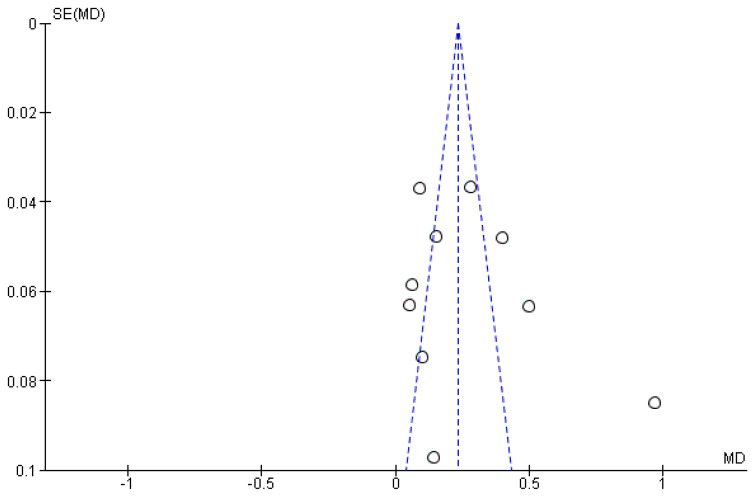
Funnel plot for the quinary outcome, *I*^2^ = 90%, was not possible to highlight a single source of data heterogeneity the study presenting the data with the greatest dispersion is that of Aguilar-Salvatierra.

**Figure 13:**
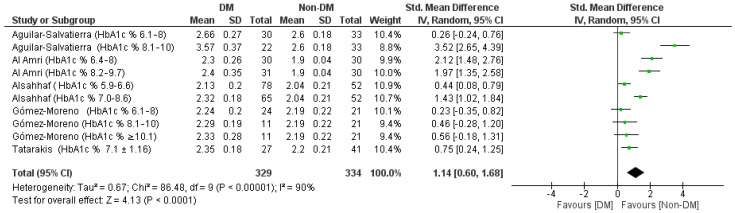
Forest plot of the random effects model of the meta-analysis of the quinary outcome.

For the aggregate Std. mean difference of the PD with a 12-month follow-up, a random effects model was applied since heterogeneity was high (*I*^2^ 90%), the analysis of the sources of heterogeneity (funnel Plot, Figure 12) and the sensitivity analysis did not reveal a single source of heterogeneity that could be selectively excluded. The results of the meta-analysis shown in the forest plot (Figure 13) were the following: 1.14 [0.60, 1.68] in favor of the non-DM group, the Tau^2^ values are 0.67 Chi² = 86.48, df = 9 (*p* < 0.00001); *I*^2^ = 90%; test for overall effect: Z = 13 (*p* < 0.0001).

**Figure 14:**
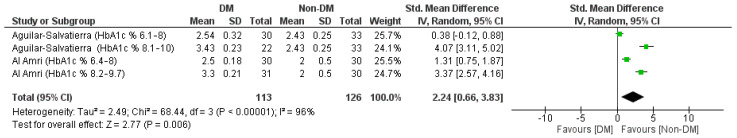
Forest plot of the random effects model of the meta-analysis of the senary outcome.

Six months old, the data resulting from the meta-analysis for senary outcome are in favor of the control group, a random effects analysis was applied given the high heterogeneity of the few data available (*I*^2^ 96%), the Std. mean difference aggregate was 2.24 [0.66, 3.83] the Tau^2^ values are 49 Chi² = 68.44, df = 3 (*p* < 0.00001); *I*² = 96%; test for overall effect: Z = 2.77 (*p* = 0.006), as reported in Figure 14.

### 3.7. Septenary Outcome (Std. MD between PI between DM and Non-DM Groups at 12 Months)

**Figure 15:**
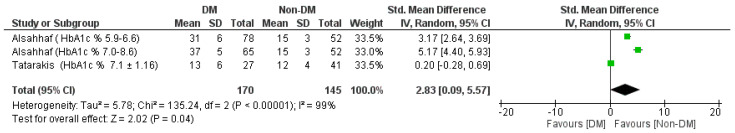
Forest plot of the random effects model of the meta-analysis of the Septenary outcome.

For the last outcome (differences in PI between diabetic and non-diabetic patient groups at 12 months) we have few data included for the meta-analysis, the results are however in favor for the non-DM group. Std. mean difference aggregate was 2.83 [0.09, 5.57] the values of Tau^2^ are 5.78, Chi² = 135.24, df = 2 (*p* < 0.00001); *I*^2^ = 99%; test for overall effect: Z = 2.02 (*p* = 0.04) (Figure 15).

### 3.8. Sub-Group Analysis

It was decided for the first, third and fifth outcome to perform a subgroup analysis according to the range and the mean of HbA1c% in the DM groups. In the first subgroup we included the data of the groups in which the patients had a HbA1c% in a range between 6 and 8, for the second subgroup the HbA1c% values had to be greater than 8.

**Figure 16:**
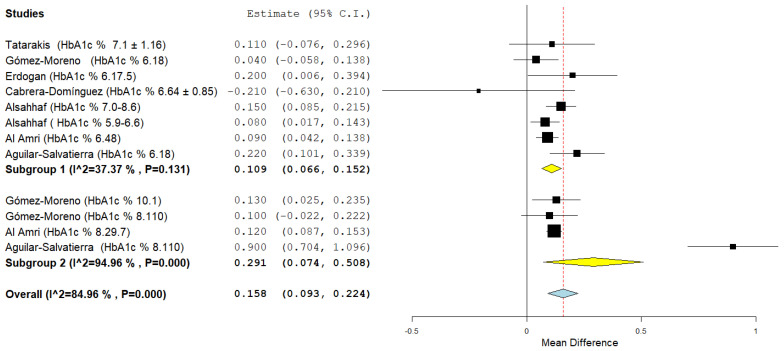
Forest plot of the subgroup analysis for the primary outcome (MBL at 12 months). Subgroup 1 (HbA1c% range: 6–8%); Subgroup 2 (HbA1c% < 8%). The results of the meta-analysis for each subgroup are highlighted in bold. Yellow rhombuses in the forest plot indicate the average effect for each subgroup investigated, the red line shows the position of the average value and the rhombus in light blue shows the measure of the average effect.

The subgroup analysis for the first outcome (Figure 16) shows a low heterogeneity for the first subgroup (HbA1c% range: 6–8%) with an aggregate mean difference of 0.109 (0.066, 0.152) *I*^2^ 37.37% and a high heterogeneity for the second subgroup (the source of the heterogeneity already investigated previously concerns the Aguilar-Salvatierra study data with values of HbA1c%: 8.1–10) *I*^2^ 94.96% and a mean difference 0.291 (0.074, 0.508), for the second subgroup the aggregate mean difference of the MBL is slightly higher.

**Figure 17:**
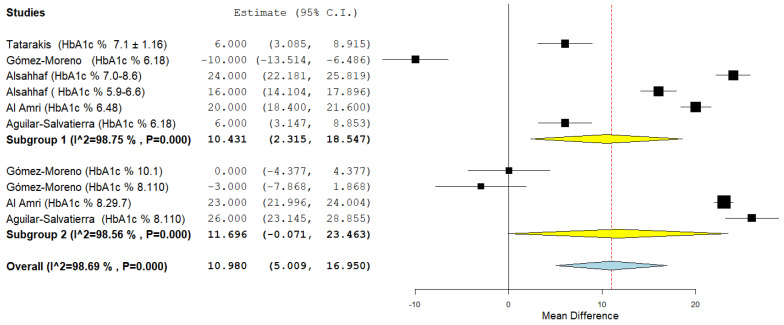
Forest plot of the subgroup analysis for the tertiary outcome (BOP at 12 months). Subgroup 1 (HbA1c%: 6–8%); Subgroup 2 (HbA1c%: <8%).

The second subgroup analysis instead concerned the tertiary outcome, heterogeneity was high and a random effects model was applied (*I*^2^ 98.75% first subgroup, and *I*^2^ 98.56% second subgroup). For the first subgroup the aggregate mean difference was 10.431 (2.315, 118.547), for the second subgroup the aggregate mean difference was 11.696 (−0.071, 23,463) (Figure 17).

**Figure 18:**
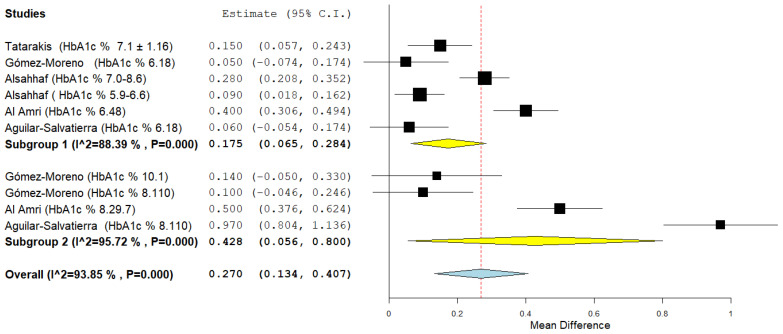
Forest plot of the subgroup analysis for the quinary outcome (PD at 12 months). Subgroup 1 (HbA1c%: 6–8%); Subgroup 2 (HbA1c%: <8%).

The last subgroup analysis concerns the quinary outcome: aggregate mean difference was 0.175 (0.065, 0.284) for the first subgroup and 0.428 (0.056, 0.800) for the second subgroup. Heterogeneity in the subgroups was high *I*^2^ 88.39% and 95.72% and a random effects model was applied (Figure 18).

The results of the meta-analyses and subgroup analyzes are, for an easier summary and representation, summarized in Table 4.

As reported before, we also used GRADE pro-GDT to evaluate the quality of the primary outcomes (Std. MD between MBL of the DM and non-DM groups at 12 months) and secondary outcomes (Std. MD between MBL in the DM and non-DM groups at 6 months) (Table 5). The results suggested that the quality of evidence is low for the first and second outcome.

## 4. Discussion

In the present systematic review, we evaluated peri-implant inflammation indices (MBL, BOP, PD, and PI) in DM patients, compared to Non-DM patients, and at the end of the selection process, totally seven studies were included in the review.

Considering the short-term (within one-year) follow-up period, the results of the meta-analysis are in support for all peri-implant inflammation indices for the non-DM group. The results of the meta-analysis for the different outcomes were found, but with less significance even when the follow-up period drops to 6 months, indicating that even in the short-term, the high blood sugar can lead to inflammatory parameters in a worse sense.

Among the studies, the difference in the identification of the groups of patients according to the levels of HbA1c%, can represent an important source of bias between the studies, in fact aggregating data from groups of patients with diabetes who have on average different HbA1c% values could distort the result. This limit could only be partially mitigated through a subgroup analysis, dividing patients with diabetes based on the range of HbA1c% values.

The analysis of the subgroups performed as a function of the HbA1c ratio, shows instead that there is no statistically significant difference between the 2 subgroups of patients with DM (First subgroup range = HbA1c% 6–8%, second subgroup range = HbA1c% < 8), but there is only a slight trend regarding the MBL and PD indices in a pejorative sense for HbA1c% < 8. 

The results of our meta-analysis partially confirm the results of previous systematic reviews of the literature: in a recent meta-analysis, Shang and Gao [33] obtain similar results for MBL 0.19 (0.08 to 0.30), PD 0.29 (0.05 to 0.52), and BOP 0.26 (0.17 to 0.35) with small differences in the two subgroups as a function of glycemic levels. Our meta-analysis completes the Shang and Gao data by extracting and comparing only the data for precise follow-up periods (Table 2) (6 months and 12 months). In fact, we performed the meta-analysis only with data corresponding to that period. Considerations on a precise period of time that previous systematic reviews had partially performed: time is a fundamental aspect if were evaluated some indices such as MBL and PD.

Furthermore, the data from a short follow-up period of six months, which had never been investigated before, confirm that even in the short term an influence of glycemic factors on inflammatory indices persists, which can contribute to implant failure over the course of a few years. Unfortunately, the subgroup analysis was not able to clearly highlight differences between the groups as a function of HbA1c levels, the only partially significant result is the subgroup analysis for the evaluation of PD and to a lesser extent MBL.

The importance of a short-term analysis of the inflammatory indices of peri-implantitis in diabetics, is especially important in light of the failure rate that implants incur in patients with diabetes already during the osseointegration phase. In this regard a recent study conducted by Tang et al. (2021) pointed out that the failure of osseointegration was present in patients with diabetes even in conditions of glycemic control compared to the control group. In fact, the authors reached the conclusion stating that glycemic control helped to limit the failure rate, but did not completely eliminate the negative effect induced by hyperglycemia during osseointegration [34].

The fundamental limitation of this review, like those present in previous reviews on this issue, is the high heterogeneity between the data provided by the studies, especially for the BOP, PD, and PI indices. The heterogeneity of the data for the MBL can be addressed through a sensitivity analysis excluding data that are source of heterogeneity and at risk of bias. In addition, the limits must also include the differences in the average age of the patients in the different groups, taking also in account that the ranges of HbA1c%, are not always uniform.

This limit can be exceeded when there are enough studies and data to be able to identify and exclude the bias within the studies and between the studies. A reason for which an evaluation was also made through the GRADE Guideline Development Tool (GRADEpro GDT) for the first two outcomes.

## 5. Conclusions

The results of the present systematic review can be of help to the clinician who has to face implant rehabilitation in the diabetic patient, because it provides data on the control and trend of peri-implant inflammatory indices in DM patients in the very short follow-up (six months).

The data provided by the meta-analysis indicate that there is already after six months a statistically significant difference in the peri-implant inflammation indices between the control group and the DM group. Consensus on the criteria for diagnosis of peri-implant diseases requires standardization of clinical and radiological outcome indicators in research. In addition, other factors that affects peri-implant health like high BMI, history of periodontal disease, oral hygiene and smoking is unequivocal but not well-researched especially in diabetic patients. Studies reported conflicting results regarding the long-term effect of diabetes on peri-implant health regardless of the level glycemic control. Therefore, interpretation of finding and relevance to clinical practice should be considered on individual bases. Future translational studies should be of longitudinal design; apply globally accepted, standard case definitions for peri-implant diseases; and monitor blood glucose levels prior to and throughout the study to provide more homogeneous, quantitative data that would allow proper comparisons between studies.

## Figures and Tables

**Table 1:** The keywords and strategy used in the SCOPUS, PubMed, and Cochrane Central Register of Con-trolled Trial databases are described in detail.

Database Provider	Key Words, Search Details	Number of Records
Pubmed	“dental implant”[All Fields] AND “diabetes”[All Fields]	176
Pubmed	((((“Diabetes Mellitus”[Mesh] OR “Diabetes Insipidus”[Mesh]) OR “Diabetes Complications”[Mesh]) OR “Diabetes Mellitus, Type 2”[Mesh]) OR “Diabetes Mellitus, Type 1”[Mesh]) AND (((((((((“Dental Implants”[Mesh] OR (“dental implants”[Mesh Terms] OR (“dental”[All Fields] AND “implants”[All Fields]) OR “dental implants”[All Fields] OR (“implants”[All Fields] AND “dental”[All Fields]) OR “implants, dental”[All Fields])) OR (“dental implants”[Mesh Terms] OR (“dental”[All Fields] AND “implants”[All Fields]) OR “dental implants”[All Fields] OR (“dental”[All Fields] AND “prosthesis”[All Fields] AND “surgical”[All Fields]))) OR (“dental implants”[Mesh Terms] OR(“dental”[All Fields] AND “implants”[All Fields]) OR “dental implants”[All Fields] OR (“surgical”[All Fields] AND “dental”[All Fields] AND “prosthesis”[All Fields]) OR “surgical dental prosthesis”[All Fields])) OR (“dental implants”[Mesh Terms] OR (“dental”[All Fields] AND “implants”[All Fields]) OR “dental implants”[All Fields] OR (“dental”[All Fields] AND “implant”[All Fields]) OR “dental implant”[All Fields])) OR (“dental implants”[Mesh Terms] OR (“dental”[All Fields] AND “implants”[All Fields]) OR “dental implants”[All Fields] OR (“implant”[All Fields] AND “dental”[All Fields]) OR “implant, dental”[All Fields])) OR (“dental implants”[Mesh Terms] OR (“dental”[All Fields] AND “implants”[All Fields]) OR “dental implants”[All Fields] OR (“dental”[All Fields] AND “prostheses”[All Fields] AND “surgical”[All Fields]))) OR (“dental implants”[Mesh Terms] OR (“dental”[All Fields] AND “implants”[All Fields]) OR “dental implants”[All Fields] OR (“surgical”[All Fields] AND “dental”[All Fields] AND “prostheses”[All Fields]))) OR (“dental implants”[Mesh Terms] OR (“dental”[All Fields] AND “implants”[All Fields]) OR “dental implants”[All Fields] OR (“prostheses”[All Fields] AND “surgical”[All Fields] AND “dental”[All Fields]))) OR (“dental implants”[Mesh Terms] OR (“dental”[All Fields] AND “implants”[All Fields]) OR “dental implants”[All Fields] OR (“prosthesis”[All Fields] AND “surgical”[All Fields] AND “dental”[All Fields]))) AND "humans"[Mesh Terms]	254
Scopus	TITLE-ABS-KEY (“dental implant” AND “diabetes”)	513
Cochrane Central Register of Controlled Trial	TITLE-ABS-KEY (“dental implant” AND “diabetes”)	49
Total		992

**Table 2:** Main data extracted from the studies.

	Study Design	Follow Up	Study Group	Mean Age (y), SD or Range	Subjects M/F	HbA1c % (Range or SD)	Number Implant Placed (Diameter/Length Expressed in mm)	Months	Plaque Index *	Probing Depth (mm)	Bleeding on Probing (%) * (Range or DS)	MBL in Millimeters (Range or DS)
[26] Tatarakis et al., 2014	Prospective	1 y	DM	65 ± 8.9 (51–80)	16 (9/7)	7.1 ± 1.16	27	12 m	0.13 ± 0.06	2.35 ± 0.18	62 ± 6	0.19 ± 0.45
			Non-DM	64 ± 8.1 (48–75)	16 (9/7)	5.7 ± 0.27	41	12 m	0.12 ± 0.04	2.20 ± 0.21	56 ± 6	0.08 ± 0.25
[27] Gómez-Moreno et al., 2015	Prospective	3 y	DM	59 ± 8.1	24 (11/13)	6.1–8	24(3.3–4.1/10–14)	12 m	\	2.24 ± 0.20	52 ± 6	0.45 ± 0.15
			DM	62 ± 6.8	11 (6/5)	8.1–10	11(3.3–4.1/10–14)	12 m	\	2.29 ± 0.18	59 ± 7	0.51 ± 0.16
			DM	64 ± 5.6	11 (7/4)	≥10.1	11 (3.3–4.1/10–14)	12 m	\	2.33 ± 0.28	62 ± 6	0.54 ± 0.12
			Non-DM	60 ± 7.2	21 (9/12)	≤6.0	21 (3.3–4.1/10–14)	12 m	\	2.19 ± 0.22	62 ± 6	0.41 ± 0.18
[28] Erdogan et al., 2015	Prospective	1 y	DM	52.6 ± 7.3	12 (5/7)	(6.1–7.5) 6.7 ± 0.3	22 (4.1/10– 12)	12 m	\	\	\	1.13 ± 0.34
			Non-DM	49.5 ± 9.3	12 (7/5)		21 (4.1/10– 12)	12 m	\	\	\	0.93 ± 0.31
[29] Aguilar-Salvatierra et al., 2016	Prospective	2 y	DM	57 ± 3.8	30 (13/17)	6.1–8	6 (3.3–12), 9 (3.3–10), 25 (4.1–14), 31 (4.1–12), 14 (4.1–10).	12 m	\	2.66 ± 0.27	45 ± 7	0.86 ± 0.25
								6 m	\	2.54 ± 0.32	0.41 ± 0.04	0.71 ± 0.31
			DM	61 ± 1.9	22 (13/9)	8.1–10		12 m	\	3.57 ± 0.37	65 ± 6	1.54 ± 0.43
								6 m	\	3.43 ± 0.23	0.59 ± 0.07	1.33 ± 0.29
			Non-DM	59 ± 2.3	33(18/15)	≤6		12 m	\	2.60 ± 0.18	39 ± 4	0.64 ± 0.23
								6 m	\	2.43 ± 0.25	0.36 ± 0.06	0.51 ± 0.19
[30] Cabrera-Domínguez et al., 2020	Prospective	2 y	DM	56.75 ± 14.76	14(9/ 5)	6.64 ± 0.85	10 (10) 4 (12)	12 m	\	\	\	0.42 ± 0.61
								6 m	\	\	\	0.28 ± 0.48
			Non-DM		14 (3/11)	5.19 ± 0.38	12 (10) 2 (12)	12 m	\	\	\	0.63 ± 0.52
								6 m	\	\	\	0.43 ± 0.40
[31] Al Amri, 2016	Prospective	2 y	DM	50.1 (46–55)	30/0	6.8% (6.4–8)	30 (10–14, 3.3–4.1)	12 m	\	2.3 ± 0.26	0.6 ± 0.04	0.54 ± 0.12
								6 m	\	2.5 ± 0.18	0.63 ± 0.06	0.52 ± 0.02
			DM	50.5 (45–59)	31	8.7% (8.2–9.7)	31 (10–14, 3.3–4.1)	12 m	\	2.4 ± 0.35	0.63 ± 0.02	0.57 ± 0.07
								6 m	\	3.3 ± 0.21	0.71 ± 0.05	0.55± 0.06
			Non-DM	48.5 (45–52)	30	4.5% (4.1–5.4)	30 (10–14, 3.3–4.1)	12 m	\	1.9 ± 0.04	0.4 ± 0.02	0.45 ± 0.06
								6 m	\	2 ± 0.5	0.42 ± 0.05	0.33 ± 0.1
[32] Alsahhaf 2019	Prospective	3 y	DM	46.1 (38–51)	41 (28/13)	6.2 (5.9–6.6)	78 (3.3)	12 m	0.31 ± 0.06	2.13 ± 0.20	0.38 ± 0.07	0.51 ± 0.14
			DM	52.7 (45–58)	38 (23/15)	7.8 (7.0–8.6)	65 (3.3)	12 m	0.37 ± 0.05	2.32 ± 0.18	0.46 ± 0.06	0.58 ± 0.15
			Non-DM	43.4(33–49)	40 (25/15)	4.7 (4.5–4.9)	52 (3.3)	12 m	0.15 ± 0.03	2.04 ± 0.21	0.22 ± 0.04	0.43 ± 0.20

* BOP values were calculated from the individual studies included as dichotomous results (presence or absence of bleeding) on 4 or 6 probes around each implant and represented as the ratio between the number of sites with bleeding and sites without bleeding, mean of the BOP in the individual studies is given by the mean of this ratio with the relative standard deviation, the value can also be expressed as a percentage (just multiply the value given by the ratio by 100), in a similar way PI was calculated as the ratio between presence and absence of plaque. This methodology is in accordance with chapter 9.4.6 Combining dichotomous and continuous outcomes from the Cochrane Handbook for Systematic Reviews of Interventions.

**Table 3:** Assessment of risk of bias within the studies (Newcastle–Ottawa scale) with scores 7 to 12 = low quality, 13 to 20 = intermediate quality, and 21 to 24 = high quality.

		Selection			Comparability		Exposure		Score
References	Definition of Cases	Representativeness of Cases	Selection of Controls	Definition of Controls	Comparability of Cases and Controls on the Basis of the Design or Analysis	Ascertainment of Exposure	Same Method of Ascertainment for Cases and Controls	Nonresponse Rate	
[26] Tatarakis et al., 2014	3	2	3	3	2	2	2	3	20
[27] Gómez-Moreno et al., 2015	3	3	2	2	2	3	3	3	21
[28] Erdogan et al., 2015	3	3	3	3	2	2	3	3	22
[29] Aguilar-Salvatierra et al., 2016	3	3	3	2	3	2	3	3	22
[30] Cabrera-Domínguez et al., 2020	3	3	3	3	3	3	3	3	24
[31] Al Amri et al., 2016	3	3	2	2	3	3	3	3	22
[32] Alsahhaf et al., 2019	3	3	2	2	3	3	3	3	22

**Table 4:** Summary data resulting from meta-analysis data.

Indices	Months	Std. MD Aggregate	*I* ^2^	Subgroup Analysis *		*I* ^2^
				HbA1c% 6–8%	HbA1c% < 8%	
**MBL**	6	1.82 [0.53, 3.10]	95%	\	\	
	12	0.81 [0.45, 1.17]	81%	0.109 [0.066, 0.152]	0.291 [0.074, 0.508]	37%\94%
**BOP**	6	3.44 [1.41, 5.50]	96%	\	\	
	12	2.84 [1.34, 4.34]	98%	10.431 [2.315, 118.547]	11.696 [−0.071, 23,463]	98%\98%
**PD**	6	2.24 [0.66, 3.83]	96%	\	\	
	12	1.14 [0.60, 1.68]	90%	0.175 [0.065, 0.284]	0.428 [0.056, 0.800]	88%\95%
**PI**	12	2.83 [0.09, 5.57]	99%	\	\	

* Mean difference aggregate.

**Table 5:** Evaluation of GRADE pro GDT.

GRADE pro GDT												
Certainty Assessment							N. of Implants		Effect			
N. of Studies	Study Design	Risk of Bias	Inconsistency	Indirectness	Imprecision	Other Considerations	Patients with DM	Patients without DM	Relative (95% CI)	Absolute (95% CI)	Certainty	Importance
**12**	observational studies	not serious (a)	Serious (b)	not serious	not serious	all plausible residual confounding would suggest spurious effect, while no effect was observed	365	369	-	MD 0.12 higher (0.1 higher to 0.14 higher)	⨁⨁◯◯ Low	IMPORTANT
**4**	observational studies	not serious	serious	not serious	not serious	all plausible residual confounding would suggest spurious effect, while no effect was observed	105	107	-	MD 0.2 higher (0.17 higher to 0.23 higher)	⨁⨁◯◯ Low	IMPORTANT

(a). The sensitivity analysis reports the data from the Aguilar-Salvatierra study (HbA1c%: range: 8.1−10) as a source of heterogeneity. In fact, excluding this study, the heterogeneity drops to 46%, this raises the question of a possible bias in this study. (b). Excluding the Aguilar-Salvatierra data (HbA1c% range: 8.1−10) the heterogeneity drops to *I*^2^ 46%.

## Data Availability

Data are contained within the article.
